# KIAA1199 expression and hyaluronan degradation colocalize in multiple sclerosis lesions

**DOI:** 10.1093/glycob/cwy064

**Published:** 2018-07-31

**Authors:** Mathieu Marella, Laurence Jadin, Gilbert A Keller, Barry J Sugarman, Gregory I Frost, H Michael Shepard

**Affiliations:** 2 Drug Discovery Department, Halozyme Therapeutics, Inc., San Diego, CA, USA; 3 F1 Oncology, West Palm Beach, FL, USA; 4 Biologics21.NET Consulting, San Diego, CA, USA

**Keywords:** Astrocyte, CEMIP, hyaluronan, KIAA1199, multiple sclerosis

## Abstract

Modification of hyaluronan (HA) accumulation has been shown to play a key role in regulating inflammatory processes linked to the progression of multiple sclerosis (MS). The aim of this study was to characterize the enzymatic activity involved in HA degradation observed within focal demyelinating lesions in the experimental autoimmune encephalomyelitis (EAE) animal model. EAE was induced in 3-month-old female C57BL/6J mice by immunization with myelin oligodendrocyte glycoprotein 33–35 (MOG33–35) peptide. The mice were monitored for 21 days. Formalin-fixed paraffin-embedded tissue from control and EAE mice were labeled with an immunoadhesin against HA, antibodies against KIAA1199 and glial fibrillary acidic protein, a marker for astrocytes. In situ hybridization was conducted using a KIAA1199 nucleic acid probe. In histologic sections of spinal cord from EAE mice, abnormal HA accumulation was observed in the close vicinity of the affected areas, whereas HA was totally degraded within the focal loci of damaged tissue. KIAA1199 immunoreactivity was exclusively associated with focal loci in damaged white columns of the spinal cord. KIAA1199 was mainly expressed by activated astrocytes that invaded damaged tissue. Similar findings were observed in tissue from an MS patient. Here, we show that KIAA1199, a protein that plays a role in a HA degradation pathway independent of the canonical hyaluronidases such as PH20, is specifically expressed in tissue lesions in which HA is degraded. KIAA1199 expression by activated astrocytes may explain the focal HA degradation observed during progression of MS and could represent a possible new therapeutic target.

## Introduction

Hyaluronan (HA), a widely distributed constituent of the extracellular matrix that accumulates in connective, epithelial and nervous tissues, is a nonsulfated glycosaminoglycan to which many physiologic functions have been ascribed. The latter include cellular migration and proliferation, tissue repair, tissue hydrodynamics, cancer progression and inflammatory processes (Hascall and Karamanos 2011; Jiang et al. 2011; Shepard 2015).

Multiple sclerosis (MS) is a chronic, inflammatory, demyelinating disease of the central nervous system (CNS) affecting ~2.5 million people worldwide (Pugliatti et al. 2002). It is characterized by an autoimmune response against the fully mature oligodendrocytes that provide myelin sheaths around axons. Demyelinated axons are unable to effectively propagate action potentials, resulting in neurologic deficits. Short-term remyelination can occur in MS lesions, transiently improving symptoms. Disease progression is accompanied by chronic remyelination failure, partially because of oligodendrocyte death and the inability of oligodendrocyte precursor cells (OPCs) to differentiate into mature myelinating cells (Plemel et al. 2017).

Maturation and differentiation of oligodendrocytes occur in distinct stages. OPCs constitute a primary stage of oligodendroglial cell maturation. They are relatively small fibroblastic cells that can migrate within the tissue and express specific cellular markers including platelet-derived growth factor receptor α (PDGFR-α) and nestin. OPCs differentiate into immature oligodendrocytes, best characterized by the expression of the myelination marker, O4 and reduced motility. Once the oligodendrocyte attaches to and covers portions of the axon, it is considered a fully differentiated cell that can synthesize large amounts of myelin basic protein (MBP), a major component of the myelin sheath. A fully mature oligodendrocyte can produce the myelin sheaths for several axons (Barateiro and Fernandes 2014).

Mouse experimental autoimmune encephalomyelitis (EAE) is a widely used experimental model of human inflammatory demyelinating diseases because it recapitulates some of the events observed during MS (Steinman 1999). EAE is a CD4-positive, T-cell-mediated autoimmune disease in which, as in MS, the demyelinating lesions of the white matter exhibit abnormal accumulation of B cells, T cells, macrophages and activated astrocytes, a condition defined as astrocytosis (Boyle and McGeer 1990; Ludwin et al. 2016). Astrocytosis is one of the hallmarks of all neuropathologies and especially neuroinflammatory events. In MS, astrocytes accumulate around damaged tissue, playing fundamental roles in disease progression (Brosnan and Raine 2013). However, they may also play a protective role by enhancing the immune responses that ultimately inhibit myelin repair but can also limit CNS inflammation and support axonal regeneration (Correale and Farez 2015). Astrocyte activation leads to regional loss of the blood–brain barrier and recruitment of pro-inflammatory immune cells via chemokine production.

HA synthesis or catabolism is crucial to OPC maturation, remyelination and immune cell activation (Back et al. 2005; Sloane et al. 2010; Lee-Sayer et al. 2015; Marella et al. 2017). Accumulation of HA fragments of different molecular weights is thought to affect disease progression. Little is known about the mechanisms underlying HA degradation and resynthesis in MS, although hyaluronidase (HYAL) activity has been postulated to contribute (Preston et al. 2013).

In contrast to previously published data, PH20 was not detected in the EAE model, although substantial HA degradation was observed (Preston et al. 2013; Marella et al. 2017). Thus, we sought to understand this phenomenon. KIAA1199 protein, also known as CEMIP or HYBID, has recently been shown to bind and participate in the degradation of HA independently of the canonical HYALs (Abe et al. 2003; Yoshida et al. 2013). Based on its known properties (Yoshida et al. 2014; Chanmee et al. 2016), we hypothesized that the KIAA1199 protein could be involved in HA degradation associated with demyelinating lesions. This hypothesis was tested in the present study using the murine EAE model, with findings confirmed in human MS tissue samples.

## Results

### HA is focally degraded in damaged areas of the EAE mouse spinal cord

**Fig. 1.**
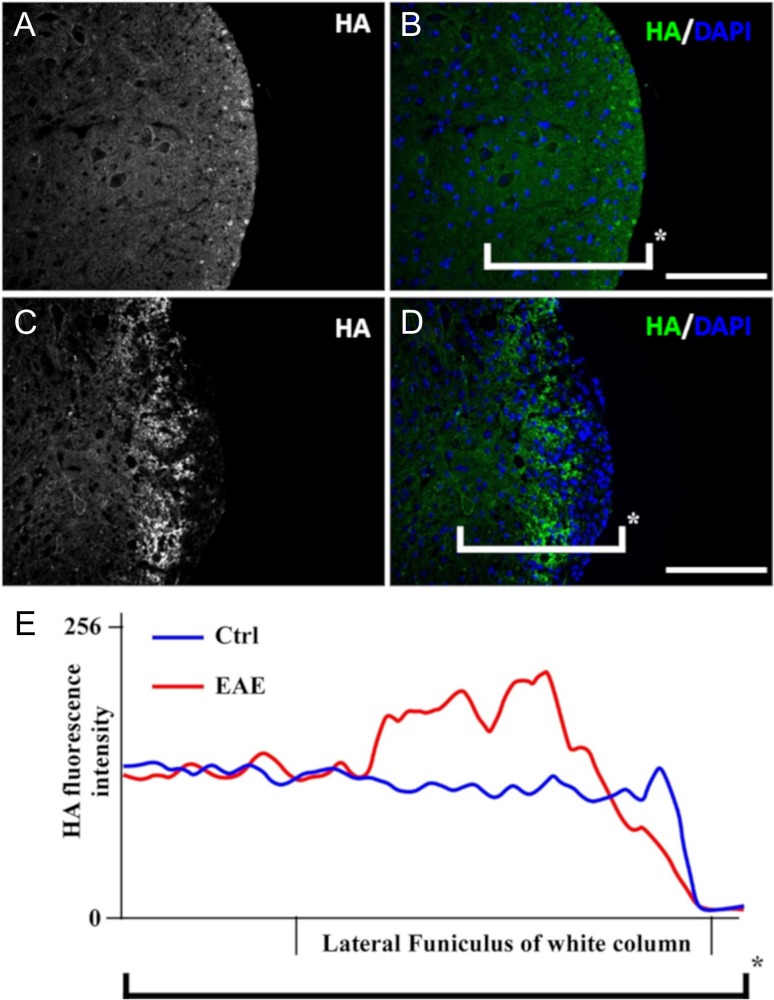
Hyaluronan (HA) distribution in the spine white matter. (**A**–**D**) 21 days after vehicle or MOG peptide injection, transverse sections of the spinal cord of control and experimental autoimmune encephalomyelitis (EAE) mice were labeled for HA. (**A**, **B**) and (**C**, **D**) are representative micrographs of control and EAE mouse, respectively. (**A**) and (**C**) show HA labeling displayed in black and white for maximum contrast with HTI-601. (**A**) and (**C**) represent HA (white) immunoreactivity within the white matter of the spinal cord of control and EAE mice, respectively. (**B**) and (**D**) are merged micrographs of HA (green) and DAPI counterstain (blue) (scale bar, 80 μm). (**E**) Distribution of the HA labeling intensity of the tissue encompassed within the brackets shown in (**B**) and (**D**).

**Fig. 2.**
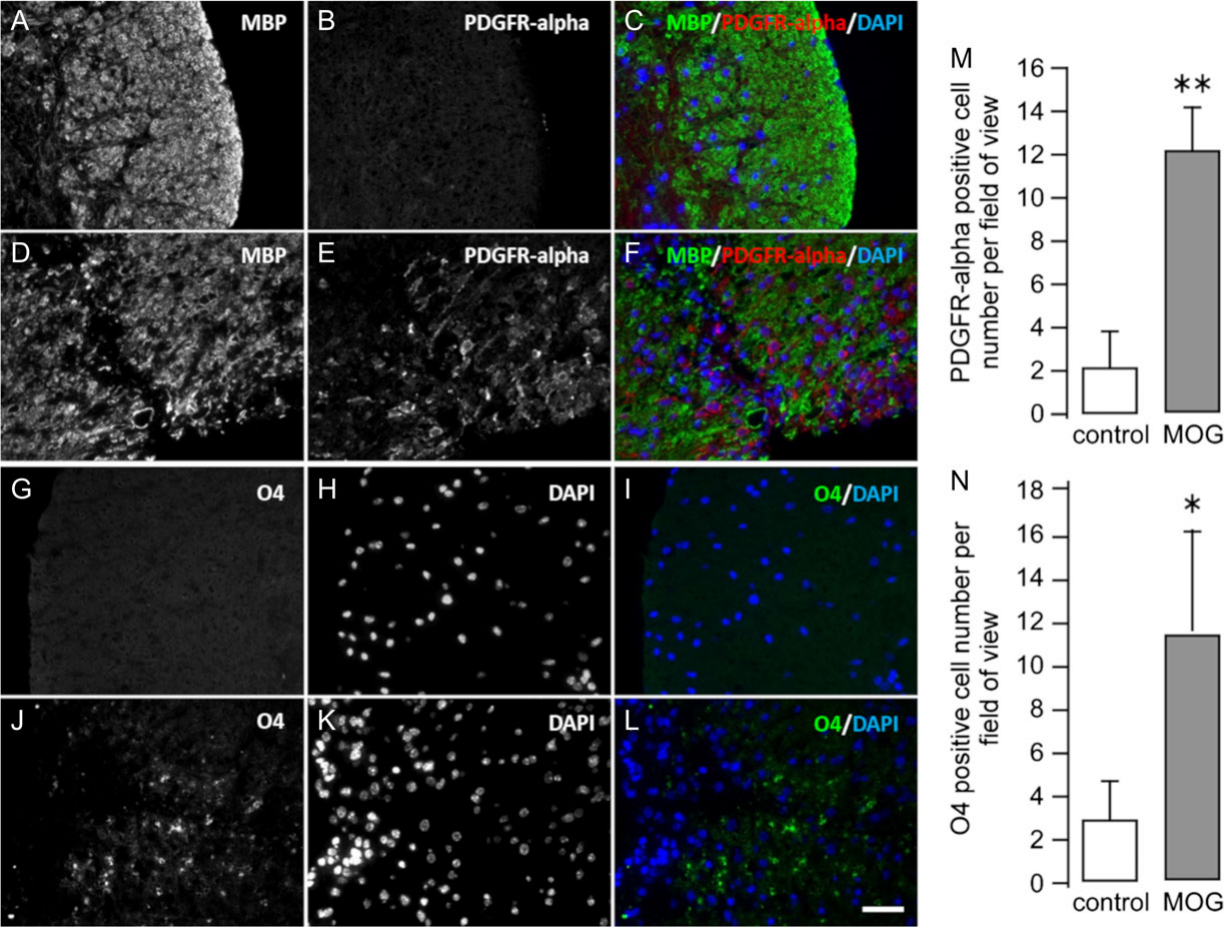
Oligodendrocytes were recruited and differentiated in the vicinity of the demyelinated areas. The different maturation steps of the oligodendrocytes were assessed in control (**A**–**C**; **G**–**I**) or EAE (**D**–**F**; **J**–**L**) mice lumbar spinal cords. Anti-platelet-derived growth factor receptor α (PDGFR-α) is expressed by oligodendrocyte precursor cells (OPCs). A high number of PDGFR-α-positive cells are labeled within the damaged areas of the EAE animal spinal cords (**F**, **M**). The positive O4 staining shows numerous premyelinated oligodendrocytes in the myelin oligodendrocyte glycoprotein (MOG)-treated spinal cord tissue (**L**, **N**). Statistical significance was evaluated by Student *t* test (*P < 0.05; **P < 0.001, compared with their respective controls, *n* = 3 control animals and *n* = 6 MOG-treated mice, three tissue sections per animal were evaluated; bar graphs represent the mean ± standard deviation).

Staining for HA was conducted on transverse sections of spinal cord from control and EAE mice. Control tissue displayed a homogeneous distribution of HA across the white matter of the section, whereas affected tissue from EAE mice showed increased HA accumulation in the proximal region of the lesion and decreased HA on the peripheral side, which coincided with the region of highest disease activity (Figure 1A–D). In sharp contrast to the distribution of HA seen in normal spinal cord (Figure 1A and B), extracellular HA appeared to be degraded within the peripheral (damaged) areas of the EAE mouse spine (Figure 1C and D), with clusters of high HA accumulation immediately adjacent to those lesions. When the HA intensity is compiled across the white column of the spinal cord, HA level in EAE mouse spines increased dramatically in the close vicinity of a damaged region but decreased abruptly at the site of cell infiltrates (Figure 1E). Transverse sections of the spinal cord of EAE mice labeled with an anti-MBP antibody showed the expected specific loss of myelination and increases in cell number in different discrete locations of the spinal cord white columns (Supplementary data, Figure S1). Myelin and HA colabeling in spinal cord sections showed a similar pattern with a strong correlation between foci of demyelination and HA degradation (Supplementary data, Figure S2). A significant increase in the number of maturing OPCs (PDGFR-α-positive) and premyelinating oligodendrocyte cells (O4-positive) was found in the surrounding damaged portions of the tissue in EAE spinal cords (Figure 2). Deficiency of HA synthase-3 (HAS-3), and to a lesser extent, HAS-1 or HAS-2, is associated with altered neuronal activity and seizures in mice (Arranz et al. 2014). Thus, we explored whether increased expression of HAS-3 could be associated with damaged areas in the EAE spinal cord. Elevated HAS3 expression, detected exclusively in remaining myelinated axons, was located in the immediate vicinity of the damaged areas in EAE spinal cord (Supplementary data, Figure S3).

### KIAA1199 protein expression was colocalized within areas of HA degradation

**Fig. 3.**
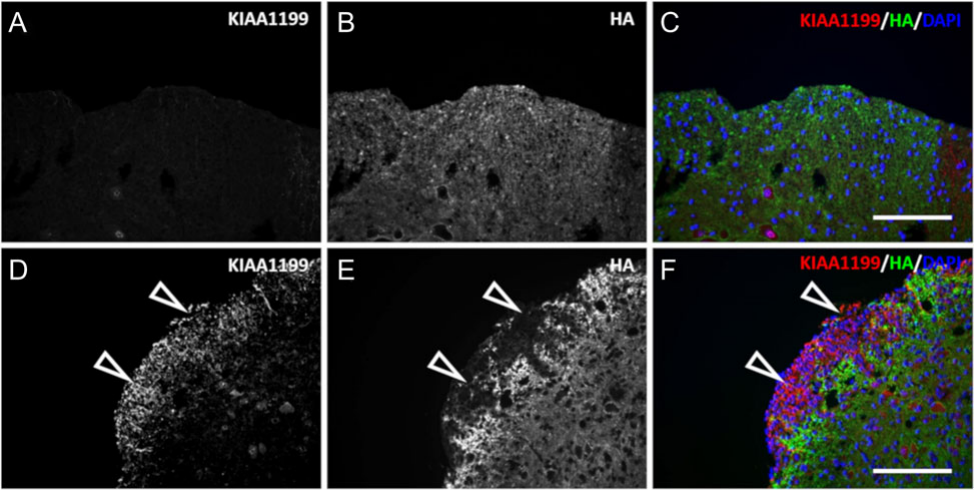
KIAA1199 is colocalized with HA-degraded areas in the EAE lesions. Transverse sections of the spinal cord of control and EAE mice were costained for KIAA1199 (**A**, **D**) and HA (**B**, **E**). (**C**) and (**F**) are the merged micrographs showing HA (green), KIAA1199 (red) and the DAPI counterstain (blue). KIAA1199 is found almost exclusively in the lesions of the EAE in which HA content is degraded (open arrows). KIAA1199 immunolabeling is mostly associated with cell structures (scale bar, 80 μm).

Others have reported that PH20 was selectively expressed in demyelinating lesions in mice with EAE (Preston et al. 2013). However, we were not able to detect PH20 expression in OPCs in normal nor demyelinated CNS of EAE mice (Marella et al. 2017). Therefore, immunofluorescence was used to determine whether a new HA-binding protein involved in HA depolymerization, KIAA1199 (Abe et al. 2003; Yoshida et al. 2013), was expressed within the damaged tissue. KIAA1199 immunoreactivity was observed only in the EAE-damaged white columns of the spinal cord (Figure 3, open arrows) in which HA was totally degraded. Control samples displayed a weak KIAA1199 nuclear staining of some motorneurons. The specificity of the positive immunolabeling was confirmed by in situ hybridization (ISH) using probes directed to portions of mRNA of the KIAA1199 gene. A clear positive correlation between the KIAA1199 probes and the anti-KIAA1199 antibody was observed in the damaged areas of the EAE spines, but no motorneuron staining was confirmed by KIAA1199 probes (Supplementary data, Figure S4). The immunolabeling for KIAA1199 was superimposable to the area of the tissue in which HA was completely degraded.

Reactive oxygen species (ROS) are soluble factors generated during inflammatory processes that can mediate HA degradation (Ågren et al. 1997). Specific by-products of oxidative reactions include 8-isoprostane, a prostaglandin-like compound produced in vivo by the free radical-catalyzed peroxidation of arachidonic acid (Praticó and FitzGerald 1996). Transverse sections of the EAE spinal cord showed a strong increase in 8-isoprostane immunolabeling, consistent with a potential role for ROS in HA degradation. No 8-isoprostane could be detected in control samples (Supplementary data, Figure S5). Nevertheless, the 8-isoprostane-positive tissue areas encompassed locations that extended beyond the discrete HA-degraded zones.

### KIAA1199 is expressed by activated astrocytes in vivo and in vitro

**Fig. 4.**
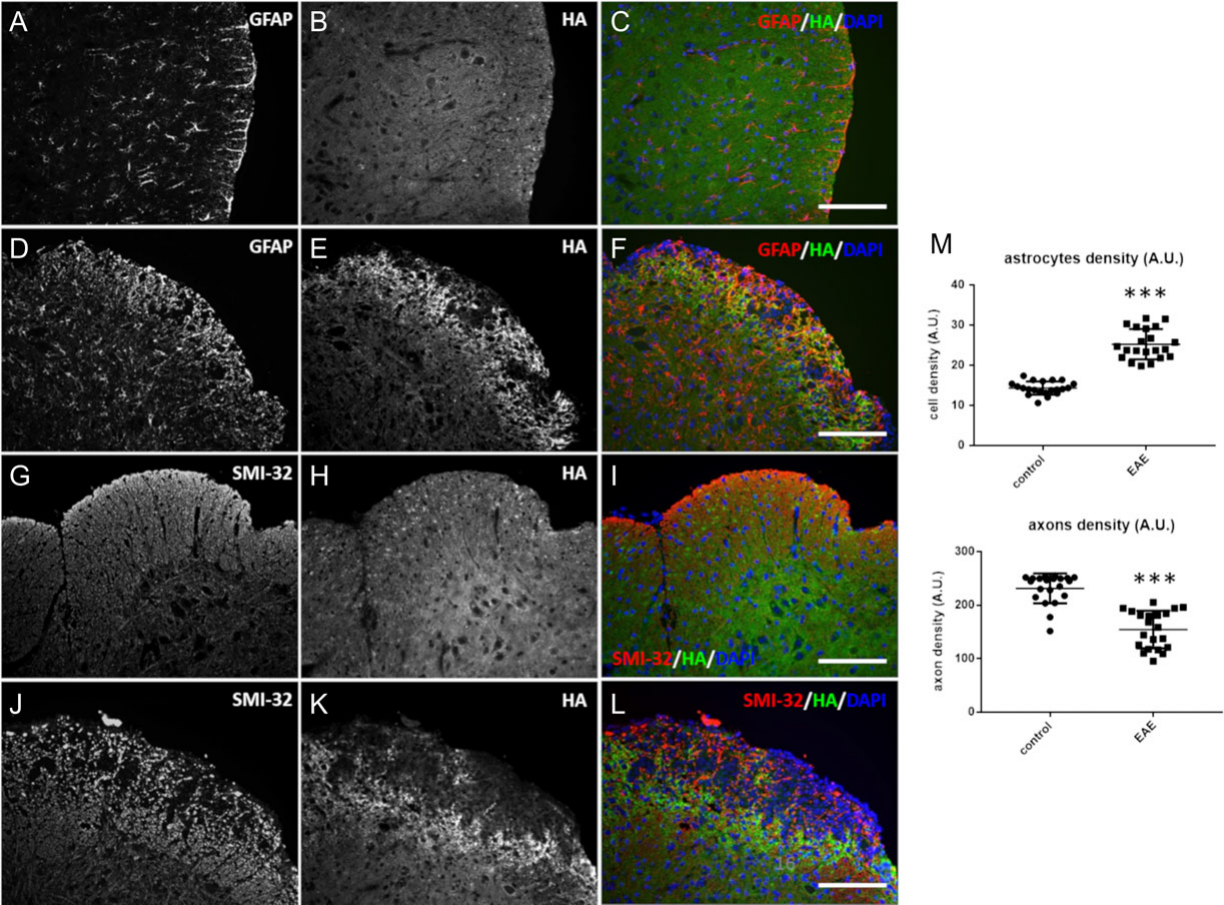
Axonal loss but not astrocytosis followed the pattern of hyaluronan (HA) degradation present in the tissue lesion. Transverse sections of the spinal cord of control (**A**–**C**, **G**–**I**) and experimental autoimmune encephalomyelitis (EAE) (**D**–**F**, **J**–**L**) mice were costained for astrocytes (GFAP) (**A**, **D**) or axons (SMI-32) (**G**, **J**) and HA (**B**, **E**, **H**, **K**). (**C**) and (**F**) are the merged micrographs that display HA (green), GFAP (red) and the DAPI counterstain (blue). (**I**) and (**L**) are the merged micrographs that display HA (green), SMI-32 (red) and the DAPI counterstain (blue). An evident astrocytosis (elevated GFAP staining) is observed in the white matter of EAE mice (scale bar, 80 μm). (**M**) shows the quantification of astrocytes and axons in the white columns of spinal cord tissue sections (seven tissue sections were evaluated for each control and EAE group. The mean along with the positive and negative standard deviations are represented. Three random fields of view for each tissue section were ascertained. ***P < 0.001, *t* test compared with respective controls).

We visualized astrocytes with an anti-glial fibrillary acidic protein (GFAP) antibody and found that their number increased in the damaged spinal cord, although their overall distribution was not restricted to the damaged areas (Figure 4A–F). Axonal loss was confined within focal portions of the tissue presenting cellular infiltrates (Figure 4G–L).

**Fig. 5.**
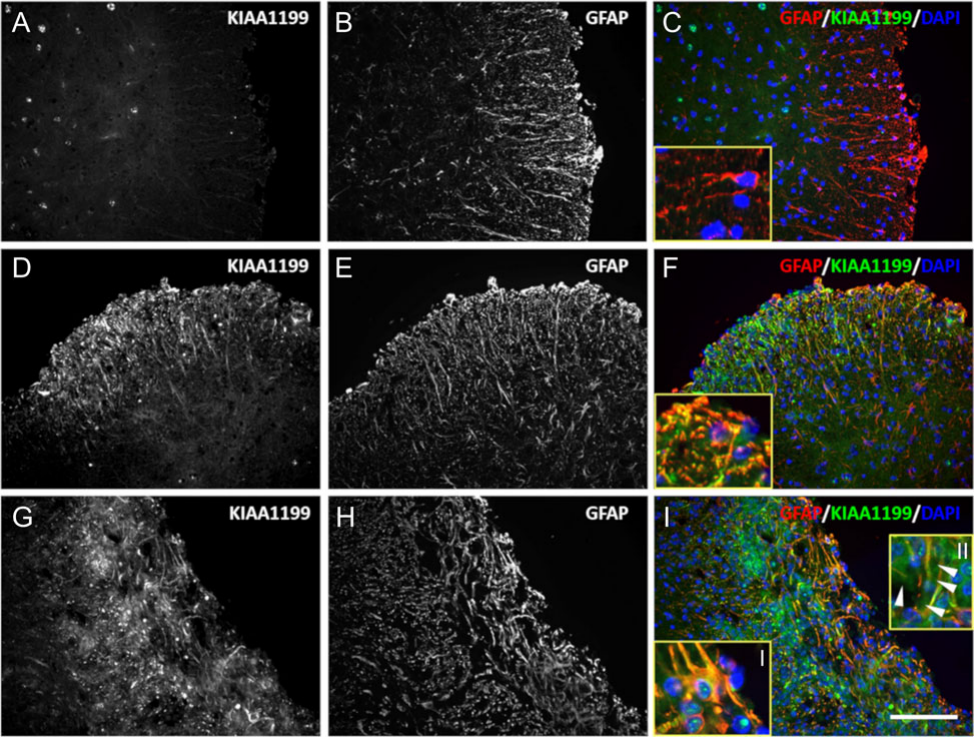
KIAA1199 protein is mainly associated with activated astrocytes. Transverse sections of the spinal cord of control (**A**–**C**) and EAE (**D**–**I**) mice were costained for KIAA1199 (**A**, **D**, **G**) and GFAP (**B**, **E**, **H**). (**C**), (**F**) and (**I**) are the merged micrographs that show KIAA1199 (green), GFAP (red) and the DAPI counterstain (blue); insets are high magnifications of the composite micrographs. KIAA1199 is mainly associated with the activated astrocyte processes. Additionally, in inset II of panel (**I**), a portion of the expressed protein does not appear to be bound to any cell structures (closed arrows) suggesting the presence of a soluble form of KIAA1199 (scale bar, 80 μm).

Confocal analysis of GFAP and KIAA1199 colabeling showed numerous areas of colocalization (Figure 5). Cell bodies and cell processes expressing KIAA1199 displayed the characteristic hypertrophic cell features of activated astrocytes (insets of Figure 5F and I). KIAA1199-positive activated astrocytes were localized in the damaged tissue of EAE mice. However, some of the KIAA1199 labeling was not associated with any cellular structure, suggesting that a soluble, extracellular form of the protein is also present in the damaged tissue near the vicinity of activated astrocytes (Figure 5I inset II).

**Fig. 6.**
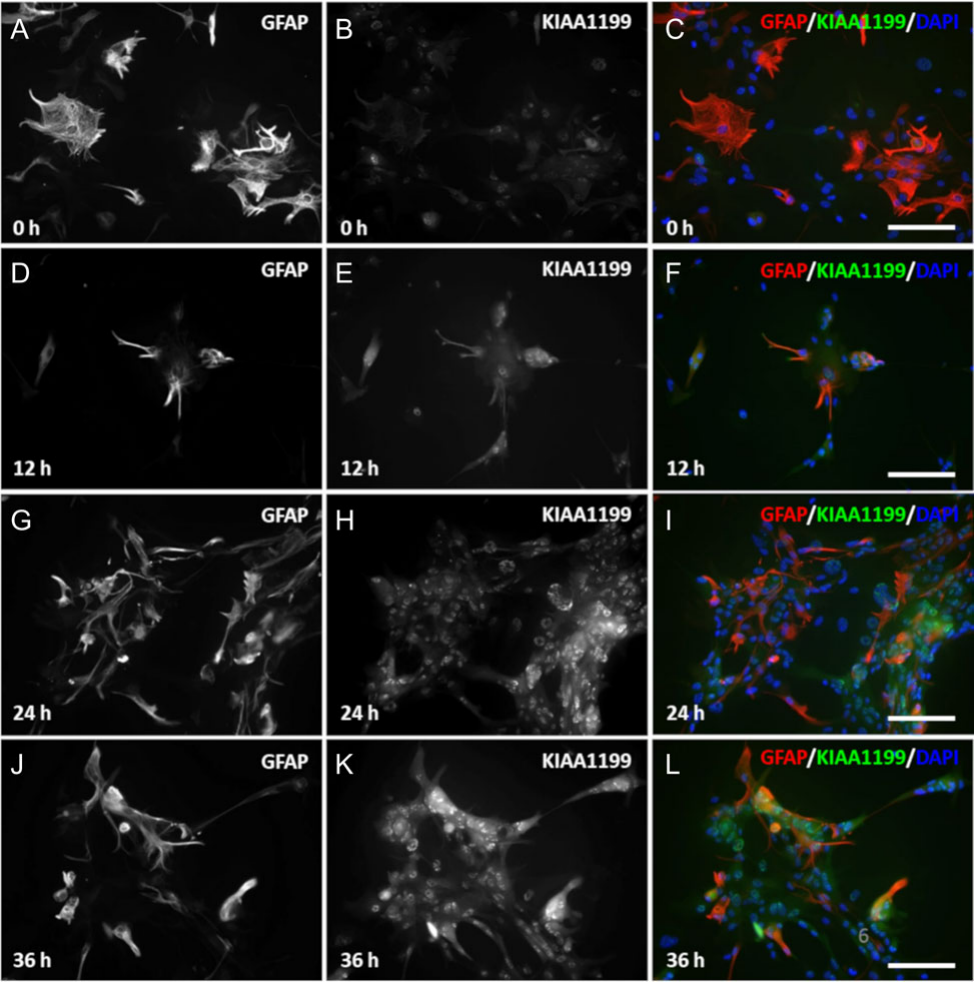
Primary mouse astrocytes incubated with a cytokine mix for 0, 12, 24 and 36 h were labeled for GFAP (**A**, **D**, **G**, **J**) and KIAA1199 (**B**, **E**, **H**, **K**). (**C**), (**F**), (**I**) and (**L**) are the merged micrographs that display KIAA1199 (green), GFAP (red) and the DAPI counterstain (blue). KIAA1199 expression was induced by the inflammatory cytokines in a time-dependent manner (scale bar, 50 μm).

To demonstrate whether KIAA1199 is expressed in activated astrocytes, a primary cell culture of cerebrum-derived astrocytes was incubated with tumor necrosis factor α (TNF-α), interleukin 1α (IL-1α) and interferon γ (IFN-γ) cytokines, and the expression of the KIAA1199 protein was assessed in vitro for up to 36 h. Within 12 h, primary astrocytes in culture began to express KIAA1199, and this expression continued to increase after 24 and 36 h of cytokine incubation (Figure 6).

**Fig. 7.**
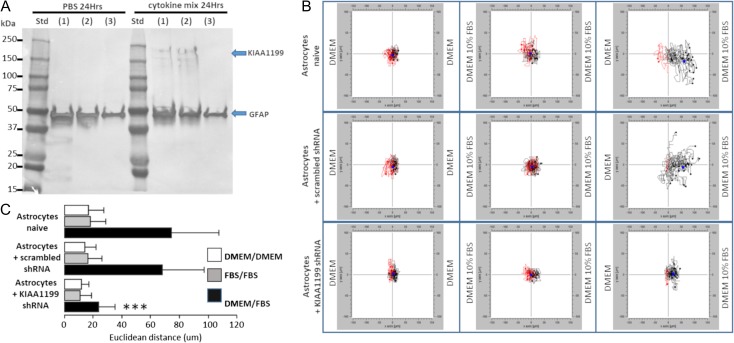
shRNA-silencing KIAA1199 expression reduces astrocyte migratory capacities through hyaluronan (HA)-rich environment. (**A**) In order to visualize KIAA1199 shRNA ability to prevent protein expression, primary mouse astrocytes were incubated or not with a cytokine mix (TNF-α, IL-1α and INF-γ) for 24 h. Expression of KIAA1199 protein from astrocyte cultures preinfected or not with KIAA1199 shRNA was assessed by western blot. (1) Naive astrocytes; (2) astrocytes infected with scrambled shRNA; (3) astrocytes infected with KIAA1199 shRNA. (**B**) Chemotaxis assays of astrocytes infected with shRNA. Astrocytes were embedded in the Hystem-C HA-rich gel and subjected or not to a 10% FBS gradient in a 3D chemotaxis chamber. Cell migration was recorded for 48 h and plotted. Astrocytes migrated toward medium containing 10% FBS. (**C**) Histogram showing the euclidean distance in micrometers migrated by the different astrocytes cultures (***P < 0.001, one-way ANOVA).

In order to assess a potential functionality of KIAA1199 expression by the astrocytes, in vitro chemotaxis assays were performed. Primary cultures of mouse astrocytes were infected by lentivirus carrying short hairpin RNA (shRNA)-targeting KIAA1199 mRNA sequences. Expression of KIAA1199 protein was assessed by western blot on culture infected or not with the scramble or specific shRNA. Figure 7 shows that KIAA1199 expression occurred when the astrocytes were incubated with a mixture of pro-inflammatory cytokines and only astrocytes infected with the anti-KIAA1199 shRNA could not express the protein under similar conditions. The same cell cultures were used in a 3D chemotaxis assay in which the astrocytes were trapped in a HA-rich scaffold (HyStem-C). The migration of those cells was induced by a gradient of fetal bovine serum (FBS) diluted at 10% in DMEM culture medium. The migration of the cells was monitored during 48 h by recording the nuclei location of 20 randomly targeted cells (Figure 7). Over a period of 48 h when exposed to the FBS gradient, the naïve astrocytes and astrocytes conditioned with scrambled shRNA migrated an average of 74.5 and 68.1 μm, respectively, representing approximately 3.5 cell lengths. Within the same conditions, the cells exposed to the lentivirus carrying KIAA1199 shRNA migrated far less: 23.8 μm representing 1.2 cell lengths. The specific inhibition of KIAA1199 reduced significantly the capacity of astrocytes to travel through this HA-rich scaffold.

### KIAA1199 expression by activated astrocytes was also observed in human MS

**Fig. 8.**
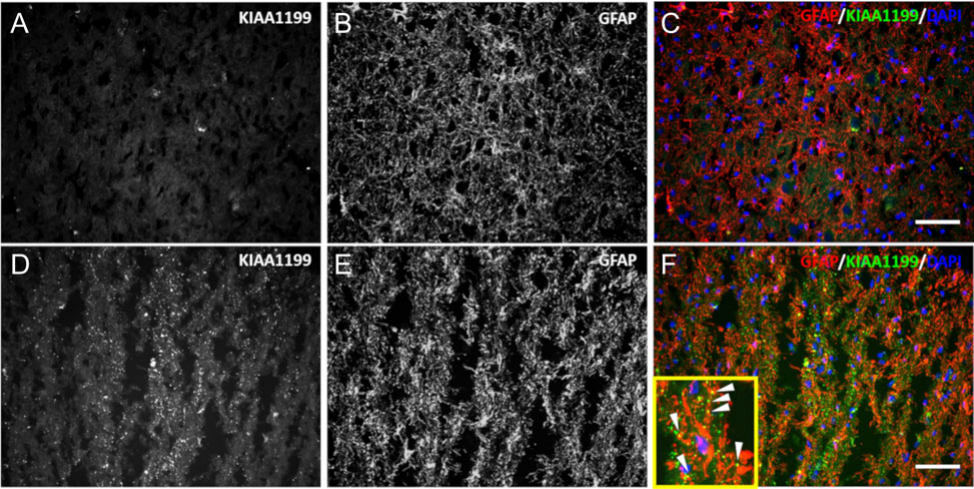
Activated astrocytes express KIAA1199 protein in the pons of an individual with multiple sclerosis (MS). Tissue sections from a control patient (**A**–**C**) or an MS patient (**D**–**F**), were costained for KIAA1199 (**A**, **D**) and GFAP (**B**, **E**). Merged panels (**C**) and (**F**) display KIAA1199 (green), GFAP (red) and the nuclei DAPI counterstain (blue). KIAA1199 expression was colocalized with GFAP-positive cells in MS samples (scale bar, 50 μm). Inset in panel (**F**) shows a high magnification of the composite image. Presence of the KIAA1199 protein within the astrocytes process is indicated by closed arrows.

**Fig. 9.**
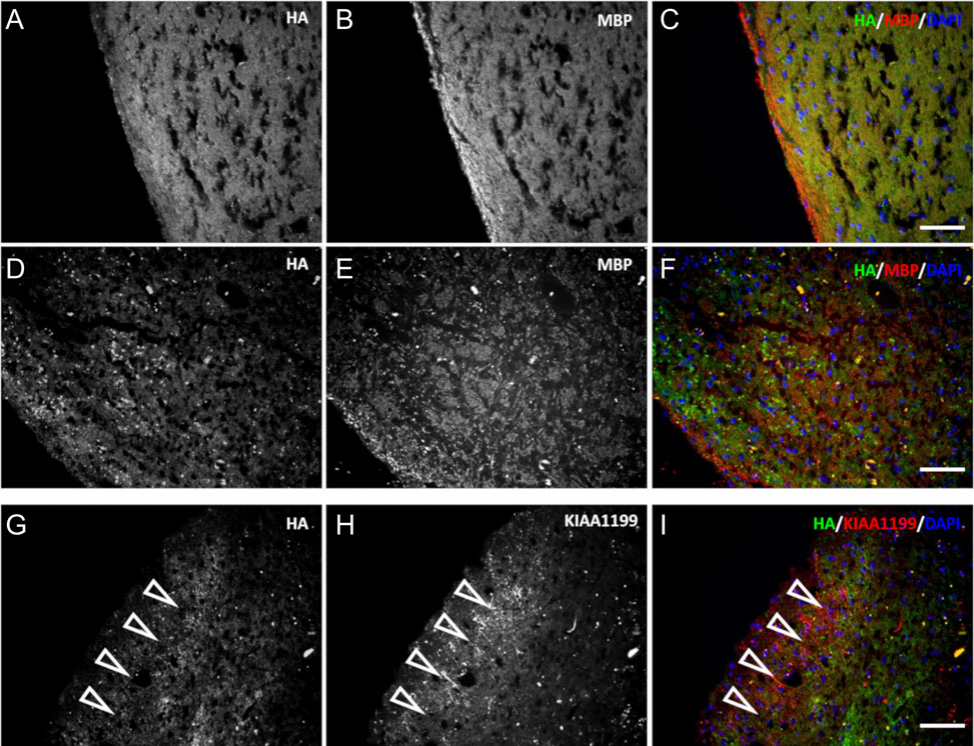
KIAA1199 protein is localized within areas of lesions depleted of HA. Tissue sections from a control patient (**A**–**C**) or an MS patient (**D**–**I**) were colabeled for HA (**A**, **D**, **G**) and myelin basic protein (MBP) (**B**, **E**) or KIAA1199 (**H**). (**C**) and (**F**) are the merged micrographs that display HA (green), MBP (red) and the nuclei DAPI counterstain (blue). (**I**) is the merged micrograph of the MS patient sample that displays HA (green), KIAA1199 (red) and the nuclei DAPI counterstain (blue). HA and KIAA1199 labeling were mutually exclusive in the tissue lesions (open arrows; scale bar, 50 μm).

Expression and localization of KIAA1199 protein was assessed in pons tissue from a normal individual and an MS patient. Sections of commercially available tissue of human pons were colabeled for KIAA1199 and GFAP protein (Figure 8). MS tissue showed strong KIAA1199 immunoreactivity (Figure 8D); control tissue did not (Figure 8A). Signs of astrocytosis, such as hypertrophic GFAP-positive cells, were present. As seen in Figure 8F, positive staining for KIAA1199 colocalized with activated astrocytes. Costaining for HA and MBP showed a uniform distribution of HA and myelinating oligodendrocytes in the control sample, and an apparent concomitant degradation of HA and demyelination in the pons of the MS patient sample (Figure 9A–I). Figure 9G–I depicts representative micrographs of an affected portion of MS tissue sample in which KIAA1199-positive staining, as in the EAE spinal cord, was seen (open arrows). The expression of KIAA1199 was superimposable to a portion of the tissue devoid of HA.

## Discussion

There was a dramatic change in HA distribution around and within focal lesions characteristic of MS damage in both human and EAE mice as compared with the respective control samples. In controls and nonaffected samples, HA was distributed homogenously within the tissue. In contrast, a large amount of HA accumulated proximal to the immediate vicinity of the tissue lesions, while none was observed within the focal damage in the EAE mouse spinal tissue. One major feature linked to MS progression is the inability of oligodendrocytes to generate new myelin sheets. In EAE mice, we observed numerous PDGFR-α-positive OPCs near the damaged areas, while only a few PDGF-α-positive OPCs were observed in the white columns of control spinal cords. HAS-3 was overexpressed within the axonal processes proximal to the damaged areas and colocalized with high HA accumulation (Supplementary data, Figure S3). This strong accumulation of HA around the damaged areas (Figures 1 and 3) could act as a physical barrier, circumventing the ongoing inflammatory processes to the least minimum space. High-molecular weight HA has been linked to anti-inflammatory effects through regulatory T cells (Itano et al. 1999; Bollyky et al. 2007).

Piao et al. (2013) reported that the HA receptor CD44 was expressed by OPCs and involved in the in vitro migration of those cells toward sites of high HA concentration, like those surrounding damaged lesions in the MS and EAE tissues in this study. Moreover, the authors reported that the loss of the HA–CD44 interaction resulted in the inhibition of OPC migration. Interestingly, we and others have demonstrated that OPC differentiation was inhibited by the presence of HA fragments (Back et al. 2005; Sloane et al. 2010; Marella et al. 2017). Therefore, the HA depolymerization and clearance that we observed within the damaged areas possibly inhibits OPC migration and contributes to their differentiation into myelinating cells.

Although it is generally accepted that HA is degraded in chronic demyelinated MS lesions and in acute lesions in EAE mice, the factors regulating this degradation remain poorly understood. Soltés et al. reported that ROS could result in HA cleavage (Šoltés et al. 2006; Stern et al. 2007). In the EAE mice spinal cords, the presence of oxidative damage near tissue lesions indicates that ROS could have played a role in the observed HA depolymerization. However, the extent of 8-isoprostane-positive tissue did not match that of the focal HA-degraded areas. Conflicting results have been reported regarding the potential role of PH20 in MS models (Preston et al. 2013; Marella et al. 2017). Here, we demonstrated that both the KIAA1199 mRNA and protein colocalized almost exclusively within the damaged regions of the EAE spinal cord where HA was degraded and were expressed mostly by activated astrocytes. These results agree with the ascribed function of KIAA1199 to bind to high-molecular weight HA and mediate HA internalization and depolymerization (Yoshida et al. 2013, 2014; Chanmee et al. 2016).

A growing body of evidence links the presence of KIAA1199 protein to inflammatory events. Soroosh et al. (2016) described the action of KIAA1199 overexpression in Crohn’s disease fibroblasts, while others have reported that the pro-inflammatory NF-kB pathway could positively influence the expression of KIAA1199 (Tak and Firestein 2001; Shostak et al. 2014). Here, we showed that KIAA1199 expression by astrocytes in vitro depends on exposure to pro-inflammatory cytokines. In vitro migration assays were carried out to characterize the physiologic advantage of KIAA1199 expression. Astrocytes appear to use KIAA1199 expression to increase their migration through HA-rich scaffolds. It has been reported that astrocytes could play opposing roles at sites of MS lesions by engaging in a protective phenotype or by exacerbating ongoing neuroinflammation (Correale and Farez 2015). Further research is needed to determine whether a therapeutic intervention aimed at decreasing KIAA1199 would help promote remyelination, as KIAA1199-driven HA degradation may be a key event promoting OPC maturation and enhancing myelin repair in MS.

## Materials and methods

### Induction of EAE in mice

EAE was induced and monitored for 21 days in 3-month-old female C57BL/6J mice (Jackson Laboratories, Bar Harbor, MA) by immunization with myelin oligodendrocyte glycoprotein 33–35 (MOG_33–35_) peptide (Hooke Laboratories, Lawrence, MA). On experimental day 0, the mice received two subcutaneous injections of 100 μL each of a solution of 1 mg/mL MOG_33–35_ peptide emulsified in complete Freund’s adjuvant. An intraperitoneal dose of 200 ng pertussis toxin was administered within 2 h of the MOG_33–35_ peptide injection and another was administered 24 h later. EAE symptoms were evaluated daily during study days 8–21. At day 21, all MOG-injected mice presented partial to complete hind limb paralysis (mice were no longer able to maintain posture of the rump and/or presented complete loss of movement of the hind limb).

### Immunofluorescence labeling

Tissue samples were harvested from control and EAE mice at study day 21 and processed to formalin-fixed paraffin-embedded (FFPE) tissue blocks. Five-micrometer sections were labeled with antibodies after ethanol-based rehydration and heat-mediated antigen retrieval. Endogenous peroxidase activity was inhibited by incubating samples in an aqueous solution of 3% H_2_O_2_ for 20 min at ambient temperature. Tissue sections were blocked in phosphate-buffered saline (PBS) containing 10% goat serum for 30 min at ambient temperature. Primary antibodies were incubated overnight at 4°C in PBS containing 10% goat serum: anti-mouse MBP (Covance, Princeton, NJ; [SMI-94R], 1/1000); rabbit anti-PDGFR-α (LSBio, Seattle, WA; [LS-C414372], 14 μg/mL); mouse anti-O4 (Chemicon/Millipore, Burlington, MA; [MAB345], 1/100); rabbit anti-HAS3 (Santa Cruz Biotechnology, Dallas, TX; H64 [SC-66917], 1/100); anti-HA immunoadhesin (Halozyme Therapeutics, San Diego, CA; [HTI-601], 1/600) (Jadin et al. 2014); goat anti-8-epi-PGF2α (8-isoprostane) (Oxford Biomedical Research, Rochester Hills, MI; [IS20], 1/300); rabbit anti-KIAA1199 (Aviva BioSystems, San Diego, CA; [ARP42526.P050], 1/400); mouse anti-neurofilament (SMI-32) (Abcam, Cambridge, UK; [Ab24574], 1/300); mouse anti-GFAP (Chemicon/Millipore, Burlington, MA; [MAB360], 1/300). After three washes of 3 min each with PBS containing Tween-20 0.05%, secondary antibodies (conjugated to Alexa Fluor^®^488 or Alexa Fluor^®^594) were applied to detect immunolabeling. Slides were mounted in Vectashield plus DAPI mounting medium (Vector Laboratories, Burlingame, CA). Representative photomicrographs were digitally captured with 5×, 20× and 40× objectives using an Axioskop microscope (Carl Zeiss, Oberkochen, Germany) equipped with a SPOT Pursuit™ camera (SPOT Imaging Solutions, Sterling Heights, MI).

### In situ hybridization

Tissue was sectioned from FFPE blocks and used within 48 h. ISH was performed using an RNAscope 2.5 HD Assay-RED (ACDBio, Newark CA; [310036]) according to manufacturer instructions. The KIAA1199 nucleic acid probe used was specific for mouse CEMIP (Mm-Cemip, ACDBio [438231]).

### Primary astrocytes shRNA transfection and migration assay

Primary astrocyte cells were generated from newborn C57BL6 mice meninges-free cortices (Marella and Chabry 2004). Astrocyte cultures contained >99% GFAP-positive cells. In order to “silence” KIAA1199 expression, primary astrocyte cell cultures were infected with batches of KIAA1199 shRNA lentivirus (Biosettia, San Diego, CA) as follows: 100 μL of each four batches of lentivirus carrying KIAA1199 shRNA (Table I) were mixed in 15 mL of culture medium and incubated with 10 × 10^6^ cells for 2 days. A total of 400 μL of the shRNA, generated by a random permutation of the CEMIP consensus sequence, was also mixed in 15 mL culture medium and incubated with 10 × 10^6^ cells. Additionally, each lentivirus construct carried a green fluorescent protein gene (*GFP*), allowing determination of the infection rate for the different astrocyte cultures (i.e., >96%).

**Table I.** Small hairpin RNA (shRNA) encapsulated in lentivirus

Clone no.	shRNA oligo sequence	Virus titer (IU/mL)
sh-Cemip-1182	AAAAGCATGGTGAACATTTCAATTTGGATCCAAATTGAAATGTTCACCATGC	2.7 × 10^7^
sh-Cemip-2067	AAAAGGATGTTGTGGGCTATAATTTGGATCCAAATTATAGCCCACAACATCC	2.1 × 10^7^
sh-Cemip-2599	AAAAGCCATTATCCGACACTTTATTGGATCCAATAAAGTGTCGGATAATGGC	2.6 × 10^7^
sh-Cemip-3670	AAAAGGGTTGCTCTTCTTGAAATTTGGATCCAAATTTCAAGAAGAGCAACCC	1.7 × 10^7^
sh-scramble	AAAAGCTACACTATCGAGCAATTTTGGATCCAAAATTGCTCGATAGTGTAGC	2.4 × 10^7^

Naive or shRNA-infected astrocytes were cultivated for 1 week. Next, 1 × 10^5^ cells were incubated with pro-inflammatory cytokines (TNF-α, 100 ng/mL; IL-1α, 50 ng/mL; IFN-γ, 0.5 mU/mL) in a 12-well plate for 24 h. Expression of KIAA1199 was assessed by western blot using a mixture of two antibodies (anti-KIAA1199, 1/1000 and anti-GFAP, 1/1000) diluted in PBS with 5% dry, nonfat milk.

Chemotaxis assays were performed in a 3D chemotaxis chamber (Ibidi, Fitchburg, WI) against a gradient of FBS diluted in DMEM. The different astrocyte cultures were embedded in an HA-rich scaffold (HyStem-C; ESI BIO, Alameda, CA). The scaffold was made following manufacturer instructions in the following proportions: glycosil/gelin (1:1) mixture 74%, DMEM 7.5% and extra-link mix 18.5%. 1 × 10^6^ astrocytes were combined into 75 μL scaffold; 6 μL were subsequently loaded into the central slot of the chemotaxis chamber. The gel was solidified for 30 min at 37°C, after which different combinations of culture medium supplemented with or without 10% FBS were added in the lateral compartments surrounding the cells. The chemotaxis chambers were equilibrated in the cell incubator for 60 min and placed onto a heated, motorized cell culture stage of an Olympus microscope. Cell migration was monitored for 48 h by imaging each chamber every 15 min (MetaMorph Research Imaging, Sunnyvale, CA). Migration was evaluated using the ImageJ cell tracker plugin (http://rsb.info.nih.gov/ij/plugins/track/track.html) and the migration tools software (Ibidi).

### ISH in human tissue

Human tissue used in this study was obtained from a third-party vendor, who was responsible for obtaining the necessary consent.

## Supplementary Material

Supplementary Data

Supplementary Data

## Abbreviations

HA: hyaluronan
MS: multiple sclerosis
EAE: experimental autoimmune encephalomyelitis
GFAP: glial fibrillary acidic protein
MOG: myelin oligodendrocyte glycoprotein
CNS: central nervous system
OPC: oligodendrocyte precursor cell
PDGFR α: platelet-derived growth factor receptor α
MBP: myelin basic protein
HYAL: hyaluronidase
HAS-3: HA synthase-3
ROS: reactive oxygen species
TNF-α: tumor necrosis factor α
IL-1α: interleukin 1α
IFN-γ: interferon γ
FBS: fetal bovine serum
FFPE tissue: formalin-fixed paraffin-embedded tissue
PBS: phosphate-buffered saline
ISH: in situ hybridization
shRNA: short hairpin RNA
