# Supplementary material for: KIAA1199 expression and hyaluronan degradation colocalize in multiple sclerosis lesions
Source: Glycobiology. 2018 Jul 31;28(12):958–67. doi: 10.1093/glycob/cwy064 (PMC6243203; doi:10.1093/glycob/cwy064)
Supplement: Supplementary Data [file cwy064_supplementaryfigurelegends_revision_new.docx]

**Legends to Supplementary Figures**

**Supplementary Figure S1.** Twenty-one days after vehicle or MOG peptide injection, transverse sections of the spinal cord of control (A–C) and experimental autoimmune encephalomyelitis (EAE) (D–F) mice were immunolabeled for myelin basic protein (MBP). (A) and (D) show immunoreactivity for MBP within the spinal cord of control and EAE mice, respectively. (B) and (E) are merged micrographs of MBP staining (green) and DAPI counterstain (blue). (C) and (F) represent the respective high-magnification view of the white matter spinal cord. The lesion of the white matter is highlighted by open arrows (scale bar, 250 µm).

**Supplementary Figure S2.** Myelin and hyaluronan (HA) distribution in the spine white matter. (A–L) Transverse sections of the spinal cord of control and experimental autoimmune encephalomyelitis (EAE) mice were immunolabeled for myelin (A, D, G, J) and HA (B, E, H, K). (A–F) show immunoreactivity of spinal cord sections of control and (G–L) are spinal cord sections of EAE mice. (C), (F), (I), and (L) are merged micrographs of myelin basic protein (MBP) (red) and HA (green) labeling, DAPI counterstain is seen in blue (scale bar, 80 µm). Open arrows highlight the damaged areas of EAE spinal cord.

**Supplementary Figure S3.** Colabeling of HAS3 with axons (neurofilament) or myelin (myelin basic protein [MBP]) in lumbar spinal cord. Transverse (A–C, I–K) and longitudinal (D–F, L–N) sections of the spinal cord harvested from experimental autoimmune encephalomyelitis (EAE) mice were colabeled with anti-HAS3 antibody and anti-neurofilament (A–G) or anti-MBP (I–O) antibodies. The merged photomicrographs in (C), (F), and (G) were pseudo-colored: green for anti-neurofilament (SMI32) labeling, red for anti-HAS3 labeling, and blue for DAPI nuclear counterstain. High-magnification photomicrographs (G) show the colocalization of HAS3 and neurofilament (arrows). The merged photomicrographs in (K), (N), and (O) are pseudo-colored, green for anti-MBP labeling, red for anti-HAS3 labeling, and blue for the DAPI nuclei counterstain. High-magnification photomicrographs show HAS3 labeling surrounded by anti-MBP labeling (arrows). Scale bar, 80 µm.

**Supplementary Figure S4.** KIAA1199 protein and KIAA1199 mRNA localization in experimental autoimmune encephalomyelitis (EAE) lesions. Transverse sections of the spinal cord of control (A, B) and EAE (C, D) mice were stained for KIAA1199 mRNA by ISH (A, C) and KIAA1199 protein by immunohistochemistry (IHC) (B, D). KIAA1199 was found almost exclusively in the lesions of the EAE. KIAA1199 protein detected by the antibody correlated with the signal for KIAA1199-specific mRNA probes. By IHC, the positive staining of motoneurons located in the gray matter (asterisks) does not seem specific as no KIAA1199 mRNA signal was detected in those cells (scale bar, 80 µm).

**Supplementary Figure S5.** Reactive oxygen species (ROS)–damaged lipids colocalized with tissue lesions. Transverse sections of the spinal cord of control (A) and experimental autoimmune encephalomyelitis (EAE) (B) mice were labeled for ROS tissue damage by an anti–8-isoprostane antibody. EAE samples displayed a high positive staining within the damaged portion of the tissue characterized by evident cellular infiltration (scale bar, 100 µm).
