# Supplementary material for: KIAA1199 expression and hyaluronan degradation colocalize in multiple sclerosis lesions
Source: Glycobiology. 2018 Jul 31;28(12):958–67. doi: 10.1093/glycob/cwy064 (PMC6243203; doi:10.1093/glycob/cwy064)

Supplementary Figure S1

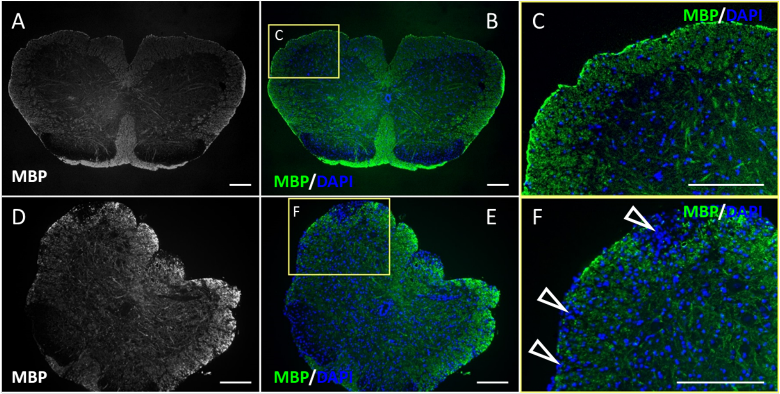

Supplementary Figure S2

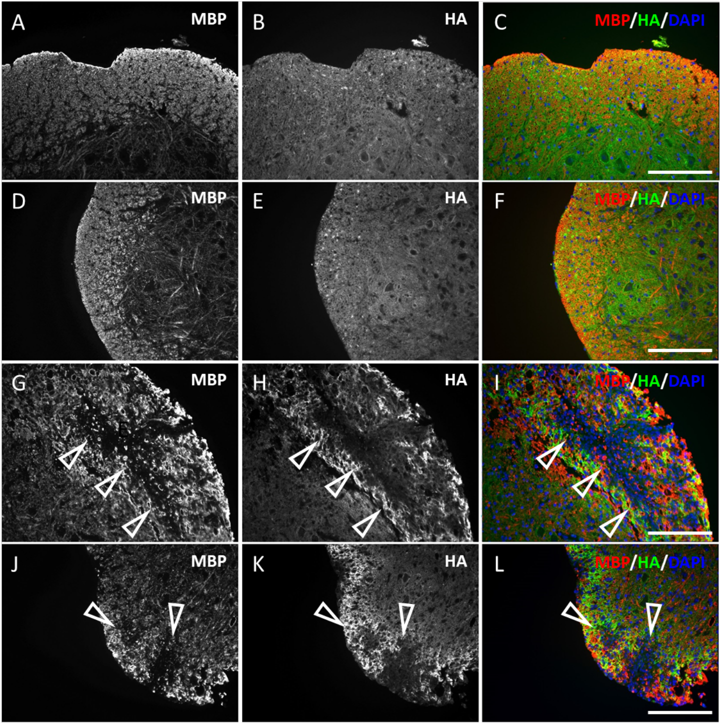

Supplementary Figure S3

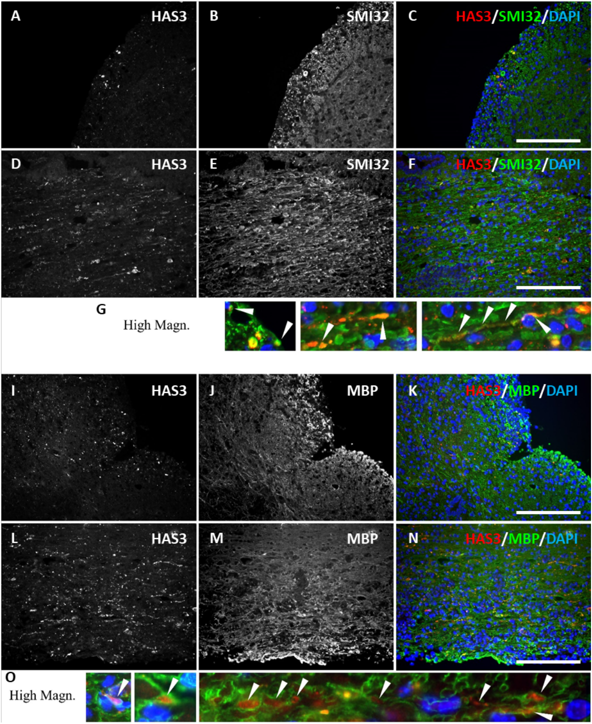

Supplementary Figure S4

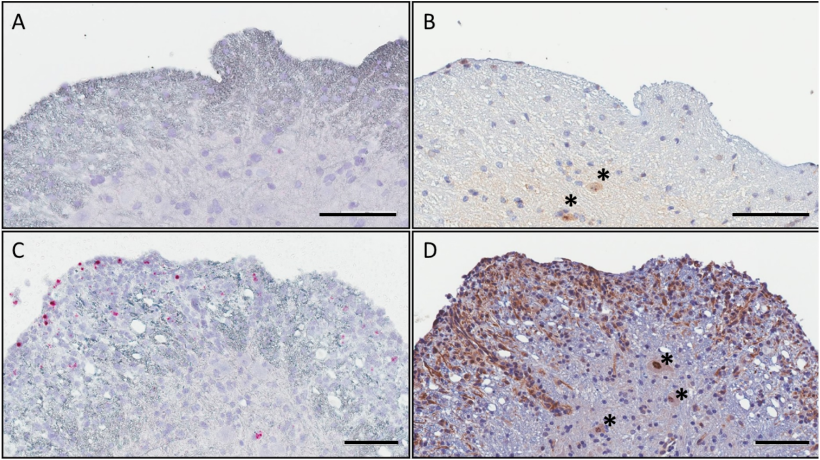

Supplementary Figure S5

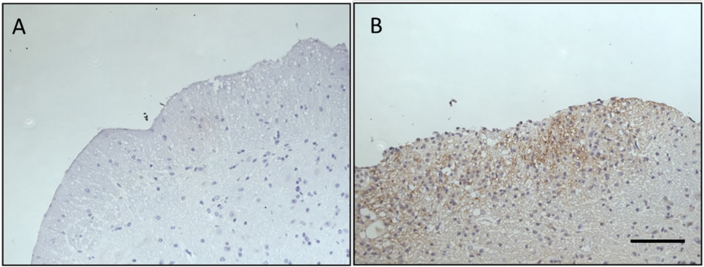

Supplement: Supplementary Data [file cwy064_supplementaryfigures_glycobiology_new_fig_order20180629.pdf]
